# Correlation Between Apparent Diffusion Coefficients and Standardized Uptake Values in Hybrid ^18^F-FDG PET/MR: Preliminary Results in Rectal Cancer

**DOI:** 10.1007/s13139-015-0390-9

**Published:** 2016-01-13

**Authors:** Ju Hye Jeong, Ihn Ho Cho, Kyung Ah Chun, Eun Jung Kong, Sang Don Kwon, Jae Hwang Kim

**Affiliations:** Department of Nuclear Medicine, Kyungpook National University Hospital, Daegu, Republic of Korea; Department of Nuclear medicine, Yeungnam University Hospital, 170, Hyeonchung-ro, Nam-gu, Daegu, 42415 Republic of Korea; Department of Surgery, Yeungnam University Hospital, Daegu, Republic of Korea

**Keywords:** Diffusion magnetic resonance imaging, Fluorodeoxyglucose F18, Multimodal Imaging, Positron-emission tomography, Rectal Neoplasms

## Abstract

**Purpose:**

Fluorine-18-fluorodeoxyglucose (^18^F-FDG) positron emission tomography (PET) and diffusion-weighted magnetic resonance imaging (DWI) share the same role in clinical oncology and it is feasible to obtain the standardized uptake value (SUV) and apparent diffusion coefficient (ADC) simultaneously by emerging the hybrid positron emission tomography/magnetic resonance (PET/MR). This study investigated the correlation between the ADCs of rectal cancer lesions and their SUVs derived from hybrid PET/MR.

**Methods:**

Nine patients with histologically proven rectal adenocarcinoma (5 men, 4 women; mean age, 70 ± 15.91 years) underwent torso ^18^F-FDG PET/CT and regional hybrid ^18^F-FDG PET/MR sequentially. A fixed threshold value of 40 % of maximum uptake was used to determine tumor volume of interest (VOI) on PET image; SUVmax, SUVpeak, and SUVmean were calculated automatically. A single freehand region of interest (ROI) was drawn on high b-value (b1000) DWI image and copied to corresponding ADC map to determine the ADCmean of rectal cancer lesion. Spearman’s rank correlation coefficient (ρ) was calculated to determine the correlation between SUVs and ADC values.

**Results:**

SUVmax, SUVpeak, and SUVmean derived by hybrid PET/MR were 12.35 ± 4.66 (mean ± standard deviation), 9.66 ± 3.15 and 7.41 ± 2.54, respectively. The ADCmean value of rectal cancer lesions was 1.02 ± 0.08 × 10^−3^mm^2^/s. ADCmean was significantly and inversely correlated with SUV values (SUVmax, ρ = −0.95, p < 0.001; SUVpeak, ρ = −0.93, p < 0.001; SUVmean, ρ = −0.91, p = 0.001).

**Conclusions:**

This preliminary hybrid PET/MR study demonstrates a significant inverse correlation exists between metabolic activity on ^18^F-FDG PET and water diffusion on DWI in rectal cancer.

## Introduction

Organ sparing procedures are preferred for the management of rectal cancer to retain sphincter, urinary, and sexual function but organ sparing is not always possible. Thus neoadjuvant therapies are currently favored for locally advanced rectal tumors. Moreover preoperative and postoperative adjuvant therapies provide benefits with respect to the control of local and regional diseases [1, 2]. The decision to apply adjuvant therapy has led to an increasing role of preoperative imaging modalities to select high-risk patients that could benefit from more aggressive treatment.

In oncology, PET/CT imaging has been successfully established as a clinical tool for tumor staging and for determining response to therapy [3]. ^18^F-FDG PET/CT provides metabolic information on tumors, based on the assumption that cancer cells generally have increased glucose utilization. SUV is a semi-quantitative measure of tissue glucose utilization in cancer and SUVmax has been widely used to quantitate the metabolic activity of tumors. Recently, an integrated whole body PET/MR scanner was introduced, and is expected to have potential value over that of PET/CT. Because MR data provide high soft tissue contrast and can provide accurate anatomical details. Furthermore, MR system can offer functional information, such as perfusion, diffusion, spectroscopy, and functional MR imaging, that would complement the metabolic information provided by PET [4]. In particular, DWI represents the functional MR techniques and has been applied in numerous cancers. DWI explores the random Brownian motion of water molecules in tissues, which is more restricted in tissues with high cellular densities, such as tumor tissues. ADC, a quantitative value derived from DWI, may provide information on biological characteristics such as tumor cellularity, tumor aggressiveness, and response to cancer treatment [5, 6] like PET SUV. In the present study, we investigated the correlation between the SUV and ADC values of rectal cancer lesions using hybrid ^18^F-FDG PET/MR to minimize the potential for physiologic changes and misregistration associated with separate PET and MR acquisitions.

## Materials and Methods

### Subjects

The study subjects were nine consecutive patients (5 men, 4 women; mean age, 70 ± 15.91 years) with newly diagnosed and pathologically confirmed rectal adenocarcinoma that underwent the torso ^18^F-FDG PET/CT, immediately followed by regional hybrid PET/MR (no additional ^18^F-FDG injection) at our institution from February 2013 to July 2013. Patients that received neoadjuvant chemotherapy or radiation therapy were excluded. Written informed consent was obtained from all patients.

### Image Acquisition

All patients fasted for at least 6 hours before the administration of ^18^F-FDG and blood glucose concentrations were confirmed to be less than 150 mg/dl. Patients received an intravenous injection of 382.91 ± 65.06 MBq of ^18^F-FDG and acquisition was started 77.56 ± 10.79 minutes later using a PET/CT scanner (Discovery VCT, GE Healthcare, Milwaukee, Wis., USA) containing bismuth germinate (BGO) crystals for PET and 64-detector row CT. Initially, a CT scan from skull base to upper thighs was obtained for attenuation correction (AC) of PET/CT images. CT parameters were as follows: 120 kV-200 mA, 3.75 mm slice thickness, 2.5 mm reconstruction thickness, and 512 × 512 matrix. A 3D mode PET scan was then performed with 7–9 bed positions at 3 minutes per bed position. The PET/CT PET scanner had an average spatial resolution of 5.0 mm at 1 cm and 5.6 mm at 10 cm from the transverse field of view (FOV) and a maximum sensitivity of 8.5 kcps/MBq at the center of the FOV.

Subsequent to obtaining torso PET/CT data, hybrid PET/MR (Biograph mMR, Siemens Healthcare, Erlangen, Germany) was performed (113.44 ± 16.51 minutes after injecting ^18^F-FDG) covering 1 bed position of the pelvis with a body surface coil (TIM, Siemens Healthcare, Erlangen, Germany). The hybrid PET/MR scanner consisted of a 3-Tesla MRI scanner with high gradient performance (maximum amplitude, 45 mT/m; maximum slew rate, 200 T/m/s) and an inline PET system with an avalanche photodiode (APD) detector. The PET scanner of the PET/MR containing lutetium oxyorthosilicate (LSO) crystals has a spatial resolution of 4.4 mm at 1 cm and of 5.2 mm at 10 cm from the transverse FOV and a sensitivity of 13.2 kcps/MBq at the center of the FOV. The PET scan was obtained for 7 minutes and MR imaging was performed simultaneously using the following sequence protocol:

1. A coronal 3D volume interpolated breath-hold examination (VIBE) T1-weighted MR sequence (repetition time (TR) 3.6 ms, echo time 1 (TE1) 1.23 ms, TE2 2.46 ms, 3.12 mm slice thickness, 4.10 × 2.60 mm in-plane resolution, 172 × 172 matrix, FOV 500 mm, generalized auto calibrating partially parallel acquisition (GRAPPA); acceleration factor 2, protocol time 19 seconds) for Dixon-based AC;
2. A coronal 3D T2-weighted Sampling Perfection with Application optimized Contrasts using different flip angle Evolutions (SPACE) (TR 1400 ms, TE 109 ms, 1 mm slice thickness, 0.98 × 0.99 mm in-plane resolution, 389 × 384 matrix, FOV 380 mm, GRAPPA acceleration factor 3, protocol time 7 minutes 11 seconds);
3. A transverse single-shot spin echo echo planar imaging (EPI) DWI with free-breathing (TR 7800 ms, TE 85 ms, b-values 0, and 1000 s/mm^2^, 3 mm slice thickness, 1.95 × 1.56 mm in-plane resolution, 115 × 160 matrix, FOV 250 mm, GRAPPA acceleration factor 2, protocol time 3 minutes 31 seconds).

A 3D ordered-subsets expectation maximization (OSEM) iterative reconstruction algorithm was applied with two iterations and 28 subsets for PET/CT PET data and with two iterations and 21 subsets for PET/MR PET data. For PET/CT and PET/MR 128 × 128 and 172 × 172 matrices were used, respectively, and in both cases PET data were filtered (6 mm full width at half maximum).

### Image Analysis

**Fig. 1:**
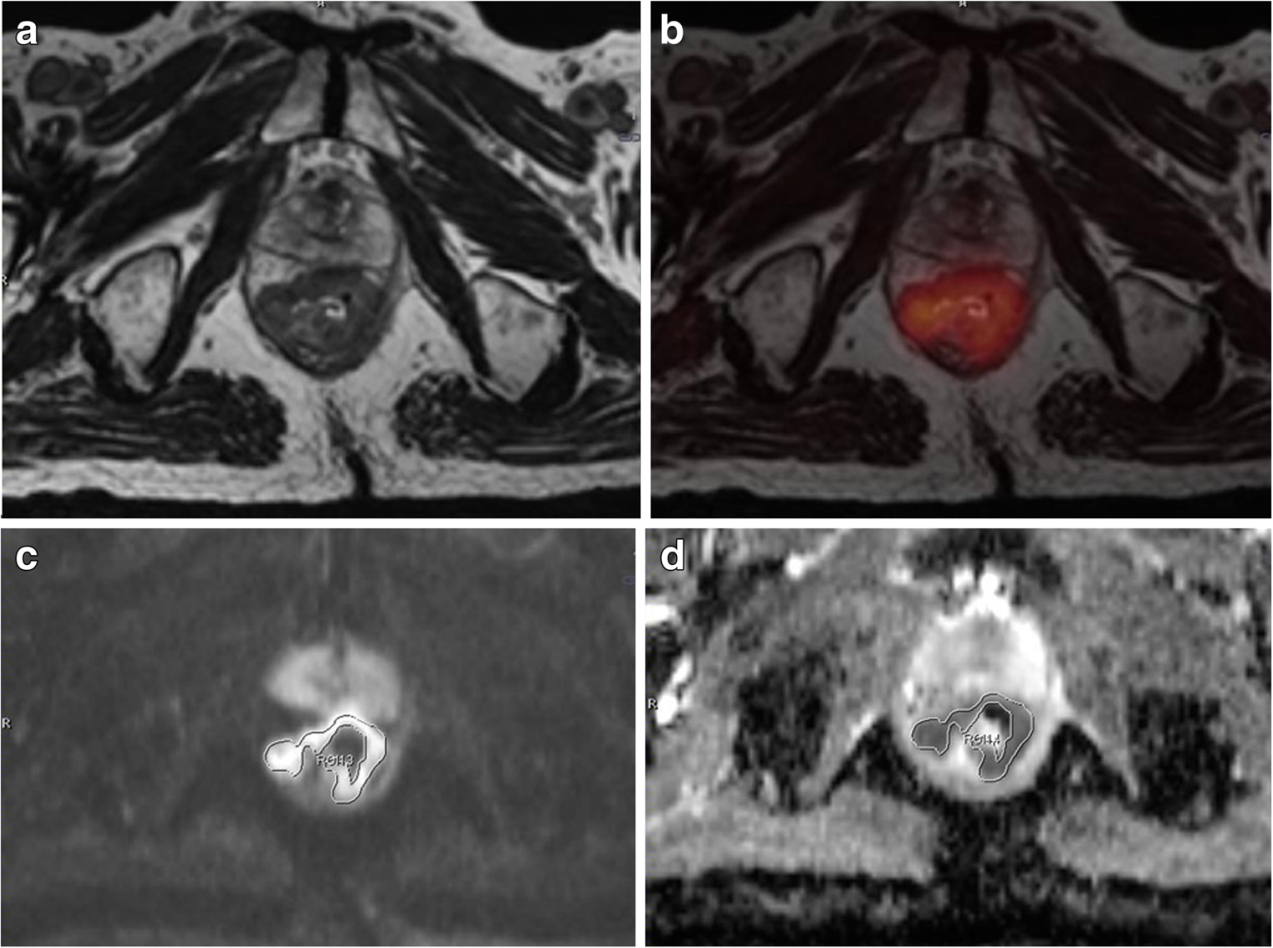
An 82-year-old man with rectal adenocarcinoma. Axial T2-weighted SPACE image (**a**), fused ^18^ F-FDG PET and axial T2-weighted SPACE images (**b**), b1000 diffusion image (**c**), and ADC map (**d**) all acquired by hybrid PET/MR. ADC value was measured by drawing a freehand ROI along the high signal intensity tumor border on a single slice b1000 image containing the largest tumor area (**c**). The ROI was copied to the ADC map (**d**) to calculate the ADCmean. ADC map was automatically generated in grayscale by the operating system, using a mono-exponential decay model

Two experienced nuclear medicine physicians interpreted PET/CT and hybrid PET/MR images using a dedicated workstation and Syngo.via software (Siemens Healthcare, Erlangen, Germany). These two readers analyzed PET/CT and PET/MR images unaware of the results of other tests and clinical information. Diagnoses were made by consensus. The maximum, peak, and mean SUVs of all rectal tumors were assessed for quantitative comparison on the PET images of both modalities. A VOI was placed over a FDG avid rectal tumor on the PET images, and an iso-contour VOI including all voxels above 40 % of maximum was then created and SUVmax, SUVpeak, and SUVmean values were calculated automatically. For ADC measurements, a polygonal ROI was manually drawn along the border of the tumor on the high b-value (b 1000) DWI image. A single freehand ROI was drawn on the single slice containing the largest tumor area and copied to the corresponding ADC map (Fig. 1). ADC maps in grayscale were automatically generated by the operating system using a mono-exponential decay model. ADCmean values were evaluated for all rectal cancer lesions.

### Statistics

The statistical analysis was performed using SPSS 21.0 software (SPSS, Chicago, Ill., USA). The Wilcoxon signed ranks test was used to determine the significances of differences between SUVs measured using the two imaging modalities. *P* values of < 0.05 were considered statistically significant. Spearman’s rank correlation coefficient (ρ) was calculated to examine the correlation between maximum, peak, and mean SUVs derived from regional hybrid PET/MR and torso PET/CT. We also evaluated the correlation between SUV and ADC values using Spearman’s rank correlation.

## Results

**Fig. 2:**
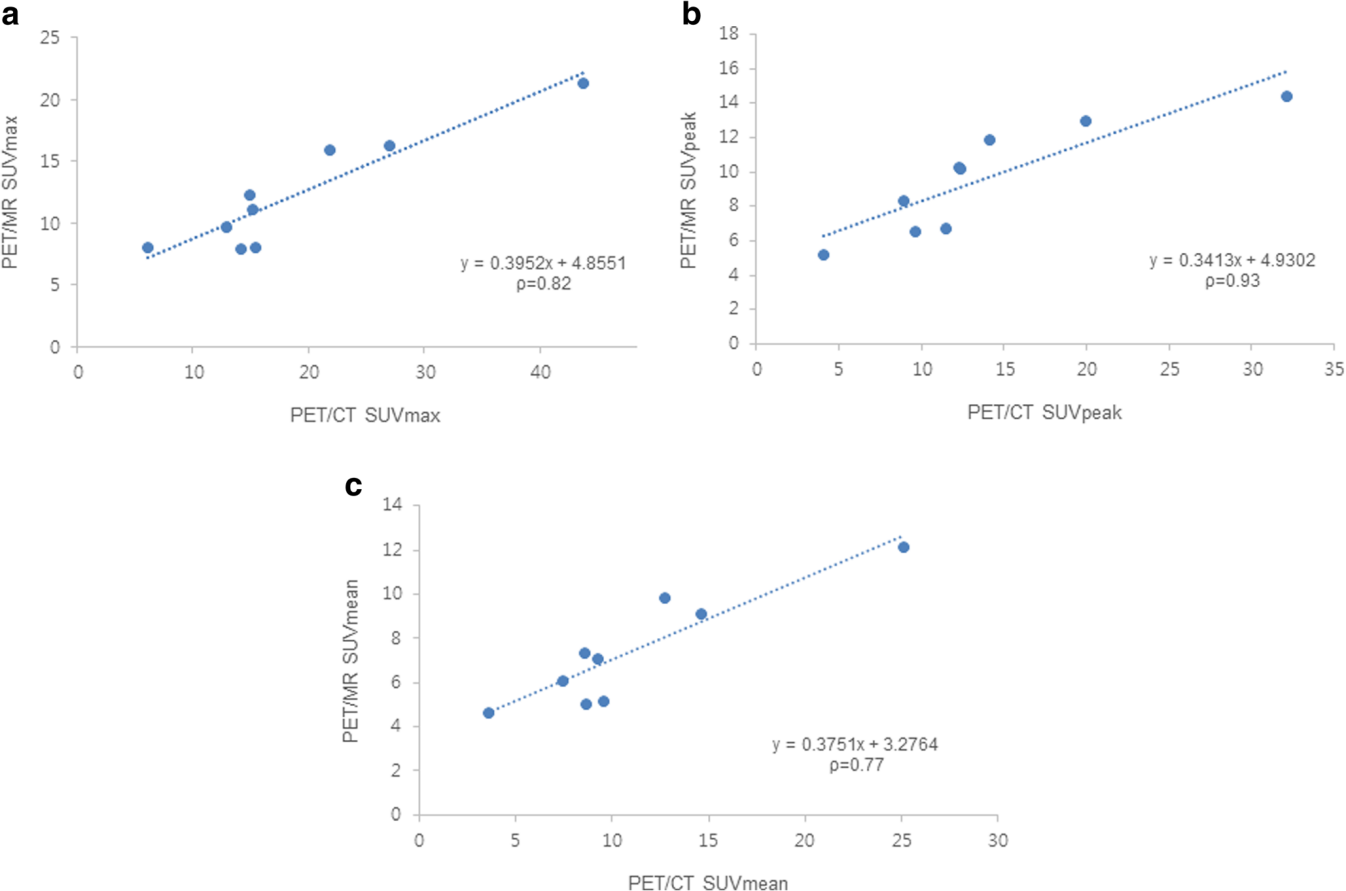
Correlation analyses of SUVmax (**a**), SUVpeak (**b**), and SUVmean (**c**) of rectal cancer lesions obtained by PET/CT and subsequent hybrid PET/MR. High correlations were found between SUV values from two modalities. ρ = Spearman’s rank correlation coefficient

For the nine rectal cancer lesions, mean SUVmax, SUVpeak, and SUVmean values obtained by hybrid PET/MR were 12.35 ± 4.66, 9.66 ± 3.15 and 7.41 ± 2.54, respectively. SUVs measured by hybrid PET/MR were significantly lower (SUVmax, p = 0.01; SUVpeak, p = 0.02; SUVmean, p = 0.01) than SUVs determined by torso PET/CT (SUVmax, 18.96 ± 10.93; SUVpeak, 13.85 ± 8.05; SUVmean, 11.03 ± 6.13). However, quantitative evaluation of PET images revealed a high correlation between maximum, peak, and mean SUVs obtained using the two modalities (SUVmax, ρ = 0.82, p = 0.007; SUVpeak, ρ = 0.93, p < 0.001; SUVmean, ρ = 0.77, p = 0.016) (Fig. 2).

**Fig. 3:**
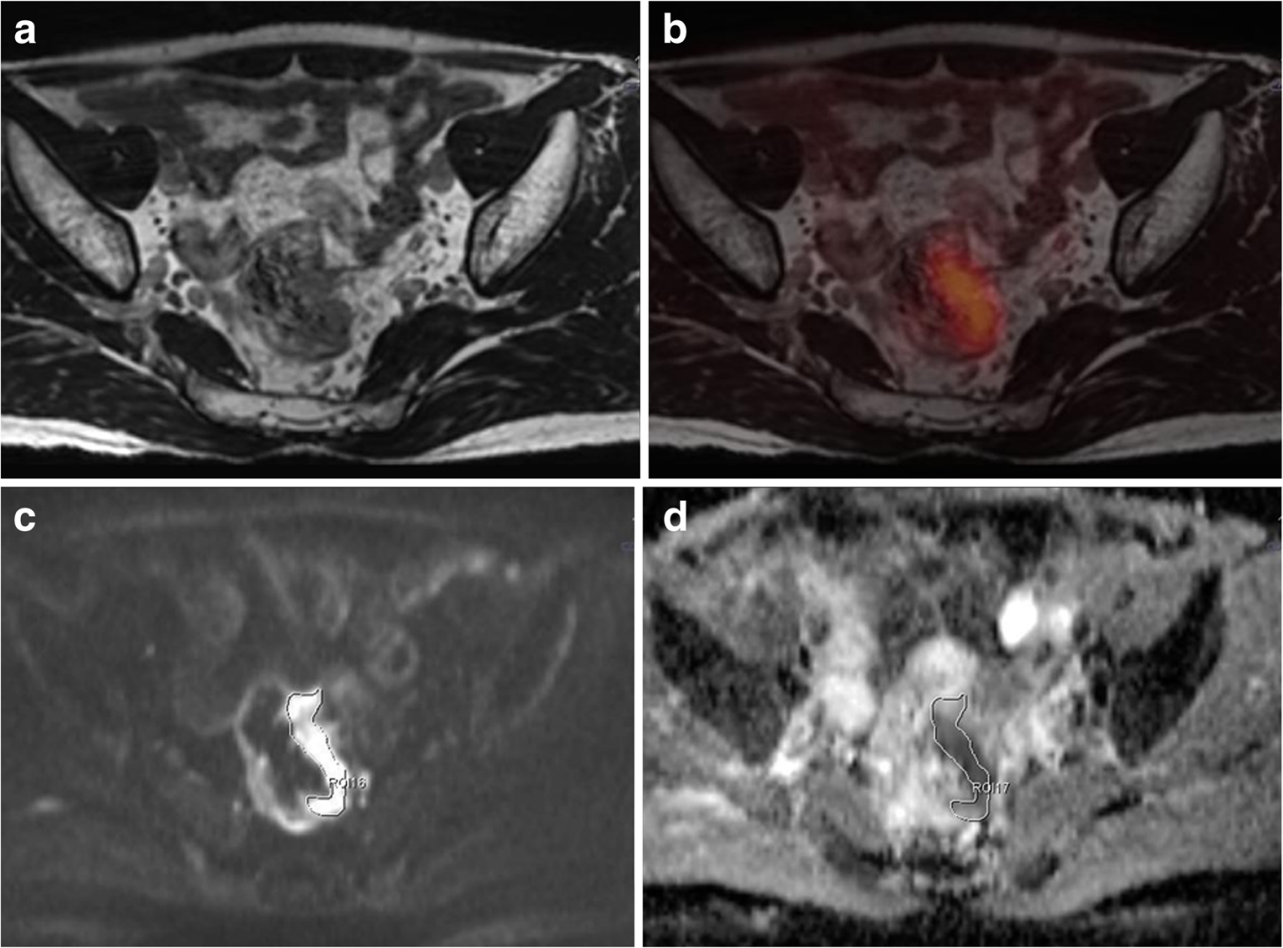
A 58-year-old man with rectal adenocarcinoma. Axial T2-weighted SPACE image (**a**), fused ^18^ F-FDG PET image and axial T2-weighted SPACE image (**b**) obtained by hybrid PET/MR showing FDG avid rectal wall thickening at the left lateral wall. SUVmax, SUVpeak, and SUVmean of the tumor were 8.14, 6.76, and 5.21, respectively. DWI (**c**) depicted the tumor as a high signal intensity lesion, and the corresponding ADC map (**d**) showed reduced ADC in the tumor (ADCmean was 1.04 × 10^−3^ mm^2^/s)

**Fig. 4:**
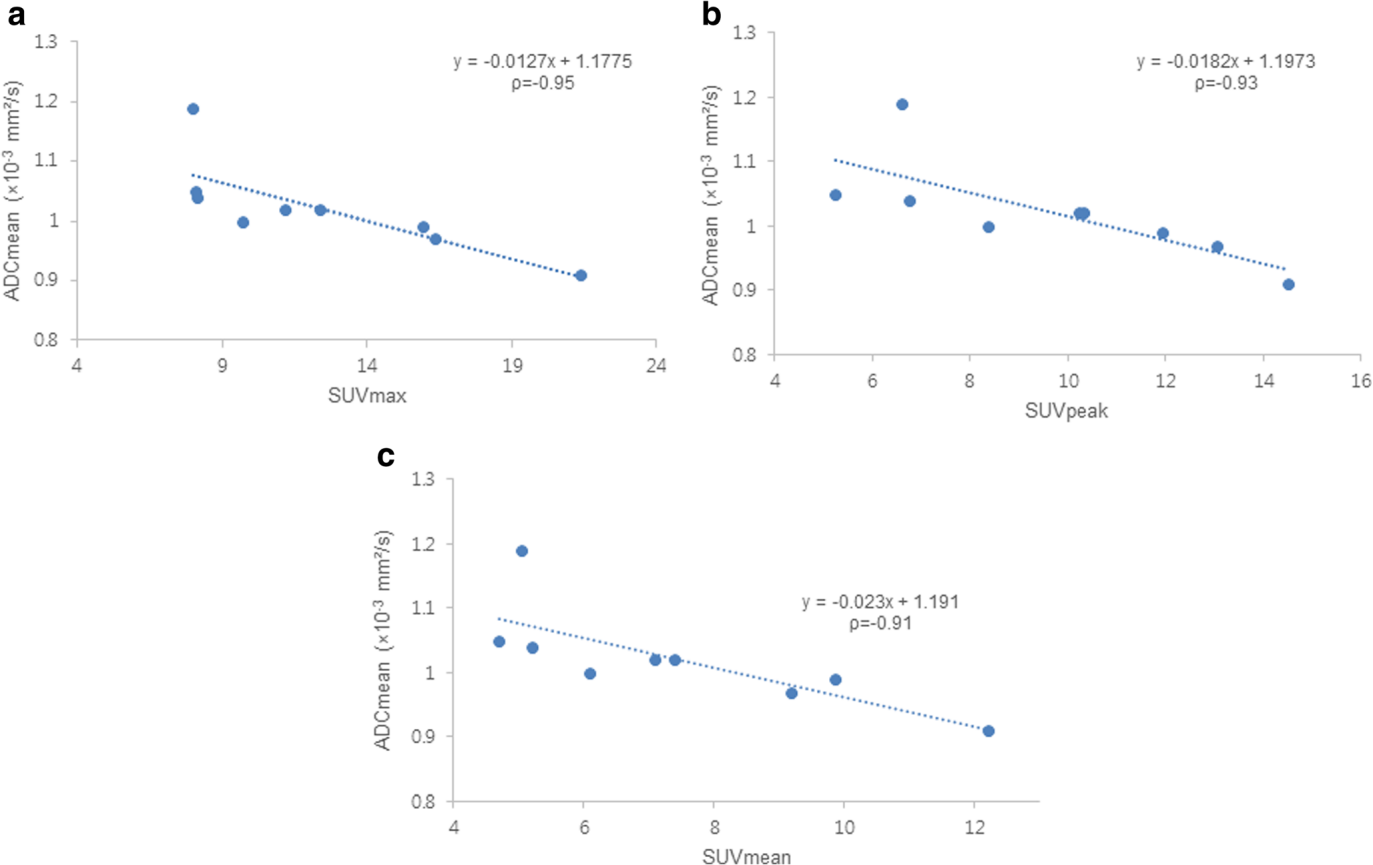
Correlations between ADC and SUV values assessed by hybrid PET/MR. Scatter plots showed the strong inverse correlations between ADCmean and SUV values. ADCmean was found to be significantly and negatively correlated with SUVmax (**a**), SUVpeak (**b**), and SUVmean (**c**) by Spearman’s rank correlation analyses (SUVmax, ρ = −0.95, p < 0.001; SUVpeak, ρ = −0.93, p < 0.001; SUVmean, ρ = −0.91, p = 0.001). ρ = Spearman’s rank correlation coefficient

Mean ADCmean value of cancer lesions was 1.02 ± 0.08 × 10^−3^mm^2^/s. Hybrid PET/MR data showed a strong inverse correlation between ADCmean and SUV values (SUVmax, ρ = −0.95, p < 0.001; SUVpeak, ρ = −0.93, p < 0.001; SUVmean, ρ = −0.91, p = 0.001) (Figs. 3 and 4). The ADCmean values of hybrid PET/MR showed a significant inverse correlation with SUVmax (ρ = −0.75, p = 0.021), SUVpeak (ρ = −0.80, p = 0.010), and SUVmean (ρ = −0.69, p = 0.041) assessed by PET/CT.

## Discussion

Hybrid PET/MR presented significantly lower SUVs than torso PET/CT for rectal cancer, which confirms recently published results [7–9]. Several factors could explain this discrepancy in SUVs. Basic technical difference, such as the AC methods used by two modalities, is probably a major cause. Whereas CT based AC in PET/CT is straightforward because CT measures the attenuation coefficients of tissues at X-ray energies, MR based AC in hybrid PET/MR is more complex because the MR signal does not provide radiodensity information. Hybrid PET/MR uses an attenuation map with a 4 tissue class segmentation (fat, soft tissue, lungs, and background/air) that is generated on the basis of a 2-point Dixon MRI sequence [10]. Several potential pitfalls of MR based AC, such as truncation artifacts resulting from insufficient coverage of arms and trunk and ignorance of attenuation by the bone, have been previously discussed [11] and these technical issues could cause discrepancy in SUV quantification. Additionally, different scanner geometries, hardware, acquisition protocols, image reconstruction algorithms, and data analyzing software are also potential technical factors. Tracer clearance with time could also explain the SUV discrepancy [9]. However, our data showed a strong correlation between SUV values of rectal cancer lesions determined by hybrid PET/MR and PET/CT (SUVmax, ρ = 0.82; SUVpeak, ρ = 0.93; SUVmean, ρ = 0.77).

Although MR based AC is a work in progress, it is expected that hybrid PET/MR will offer further advantages over PET/CT. A variety of functional information including diffusion can be added to the metabolic information provided by PET as well as morphologic information. Furthermore, simultaneous data acquisition enables real time multi-parametric functional imaging. ^18^F-FDG PET images increased glycolysis resulting from a specific metabolic abnormality of cancer cells, which is known as the Warburg effect [12]. It has been suggested that FDG uptake reflects tumor grading and cellular proliferation because less differentiated and more rapidly growing tumors probably metabolize more glucose for energy production. DWI depicts water diffusion in biologic tissue and the degree of restriction of this diffusion is inversely related to tissue cellularity and cell membrane integrity. Hence ADC is also considered a parameter of tumor characteristics such as tumor cellularity, tumor aggressiveness and cancer treatment response [5, 6]. Previous studies have reported negative correlations between SUV and ADC in pancreatic cancer, non-small cell lung cancer, head and neck squamous cell carcinoma, GIST, and rectal cancer [13–18]. In the present study, hybrid PET/MR demonstrated a strong inverse correlation between ADCmean and SUV values (SUVmax, ρ = −0.95; SUVpeak, ρ = −0.93; SUVmean, ρ = −0.91) in rectal cancer. The ADCmean values of PET/MR were also found to be significantly and negatively correlated with SUVmax (ρ = −0.75), SUVpeak (ρ = −0.80), and SUVmean (ρ = −0.69) values assessed by PET/CT, although Spearman’s rank correlation coefficients were smaller than for hybrid PET/MR. These results are in agreement with previous reports on rectal cancer [17, 18] and with expectation, because SUV and ADC values are directly related to tumor cellularity. Therefore, we suggest the significant negative correlation found between SUV and ADC probably represents a true biologic relation between tumor cellularity and metabolic activity and that these two parameters play a complementary role in terms of describing tumor characteristics, assessing treatment response, and planning treatment in rectal cancer.

SUVmax is relatively independent of tumor size and shape because tumor is segmented by adaptive thresholding, and thus, SUV measurements are rapid and highly reproducible. In contrast ADC measurements are influenced by ROI size and placement and by interobserver variability [19]. On the other hand, the simultaneous acquisition of SUV and ADC data without patient motion by hybrid PET/MR minimizes biologic changes and misregistration artifacts, and probably provides stronger negative correlations than were previously obtained by separate modalities (PET/CT and MR) with an interval of time.

Our study has some limitations. First, we assessed only ADCmean values, which are generally accepted as a more reliable indicator of tumor cellularity since the entire lesion is taken into account. Although ADCmin had been suggested to reflect highest tumor cell density or the most proliferative portion of a tumor [18], the use of ADCmin is likely to result in high errors due to the effects of lesion heterogeneity or artifacts. For these reasons, we did not evaluate ADCmin values in the present study. Second, we measured ADC value on a single slice containing the largest available tumor area. ADC measurements obtained using whole tumor volumes were more reproducible than those obtained from single-slice or small sample measurements in rectal cancer. However, there was no significant difference between the tumor ADCs obtained using whole-volume measurements and the single-slice approach in the study of Lambregts et al. [19]. Thus, we chose the single-slice method because the whole-volume ROIs protocol is time consuming and simpler, quicker methods are preferred in clinical practice. Third, only a small number of patients were included, and thus, our find should be considered preliminary. Accordingly, further studies are needed to confirm our results.

## Conclusions

This hybrid PET/MR study demonstrates a significant negative correlation exists between metabolic activity on ^18^F-FDG PET and water diffusion on DWI in rectal cancer, presumably because both parameters are directly related to tumor cellularity. The correlation found between SUVs and ADC values supports the notion that high cellularity due to tumor proliferation results in greater metabolism activity and restricts water diffusion. Further studies are needed to clarify the complementary roles of SUV and ADC with respect to the determination of tumor characteristics, the assessment of treatment response, and the planning of treatment for rectal cancer.
